# Fever of Unknown Origin in a Young Woman With Multiple Comorbidities: A Diagnostic Challenge

**DOI:** 10.7759/cureus.78361

**Published:** 2025-02-01

**Authors:** Sonal Kumar, Carlos Rincon-Vazquez, Patricia Ward, Taylor E Collignon, Taisiya Tumarinson

**Affiliations:** 1 Surgery, Ross University School of Medicine, Miramar, USA; 2 Internal Medicine, Ross University School of Medicine, Miramar, USA; 3 Internal Medicine, St. George's University School of Medicine, St. George's, GRD; 4 Internal Medicine, Lake Erie College of Osteopathic Medicine, Bradenton, USA; 5 Infectious Disease, Cleveland Clinic Florida, Weston, USA

**Keywords:** autoimmune diseases, fever of unknown origin, granulomatous hepatitis, hhv-7, liver biopsy, parvovirus b19, pituitary microadenoma, polycystic ovary syndrome, systemic lupus erythematosus, thromboembolic events

## Abstract

Fever of unknown origin (FUO) presents a diagnostic challenge, requiring detailed evaluation of infectious, autoimmune, inflammatory, and neoplastic causes. We report the case of a 28-year-old female patient with a history of polycystic ovary syndrome, pituitary microadenoma, and prior thromboembolic events, who presented with two months of persistent fever, abdominal pain, and diarrhea. The patient recently traveled to Europe and reported freshwater swimming and walking barefoot on beaches. Initial imaging showed multifocal hepatic abscesses and colitis which prompted empiric antimicrobial therapy which showed minimal improvement. Extensive infectious workup, including malaria, HIV, viral hepatitis, and parasitic testing, was negative. In terms of laboratory evaluation, the patient’s complement C3 was elevated (176 mg/dL), C4 was normal (29 mg/dL), and ANA, p-ANCA, and c-ANCA were negative. Karius testing detected HHV-7 and parvovirus B19 exposure, with equivocal Brucella IgM. Liver biopsy demonstrated non-necrotizing granulomas and abscesses, but cultures were negative. The patient ultimately responded well to therapy with corticosteroids with her fever resolving and showing signs of significant clinical improvement. This case presentation highlights the rarity of lupus-associated granulomatous hepatitis as FUO showing the importance of considering autoimmune diseases in the differential diagnosis and utilizing liver biopsy to uncover uncommon etiologies.

## Introduction

Systemic lupus erythematosus (SLE) is a complex and chronic autoimmune disease defined by multisystem involvement and presents in patients differently. Due to its ability to mimic various conditions, SLE has been referred to as the great imitator [1,2]. While some clinical features are common and overlapping such as arthritis, skin manifestations, and renal involvement, there are atypical presentations such as fever of unknown origin (FUO) that can delay diagnosis [3]. FUO is a fever for three weeks or longer without an identifiable cause despite thorough investigations and presents a diagnostic challenge for clinicians. Providers are forced to differentiate between infectious, neoplastic, and autoimmune causes, particularly in patients with persistent and unexplained fever [4,5]. Fever is a frequent symptom of SLE, occurring in approximately 36% to 86% of patients. In certain cases, it may present as the initial and sole manifestation of the disease [6]. Infections are often the first consideration in cases of FUO; however, non-infectious causes, including autoimmune diseases like SLE, must also be evaluated, particularly in young women who are disproportionately affected by SLE [4,6]. This demographic accounts for the majority of SLE cases, with peak incidence during the reproductive years [1]. Early detection and diagnosis are critical for disease management, as SLE if untreated can result in morbidity and mortality related to organ damage or further complications such as infection [3,7]. This report tells the diagnostic journey of a young woman presenting with unrelenting FUO and ultimately diagnosed with SLE. Our case highlights the need to maintain a broad differential diagnosis and addresses the need for a high index of suspicion for autoimmune conditions in patients with atypical presentations of prolonged fever.

## Case presentation

**Figure 1:**
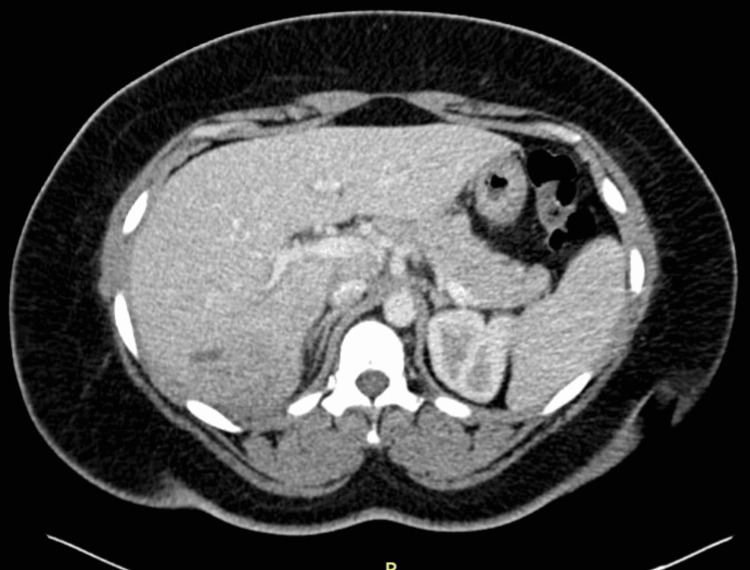
CT showing markedly abnormal appearance of the liver.

A 28-year-old woman with a history of polycystic ovary syndrome, hirsutism, cystic acne, uterine bleeding, anemia, prediabetes, hyperlipidemia, hypertension, HSV-II, obesity (BMI 46.16), thrombophilia (low antithrombin III, elevated factor VIII, PAI-1 gene mutation), and a pituitary adenoma presented to the Emergency Department (ED) with a two-week history of abdominal pain, diarrhea, fever, and chills. She also had a cholecystectomy in 2010. The patient's family history includes cardiovascular disease and clotting disorders; her mother had heart disease, hypertension, and heart failure, while her father died of liver failure. Her sister and first cousin both had pulmonary embolism. Socially, the patient used marijuana (now discontinued) and drinks one alcoholic beverage per week; she has never smoked. The patient had traveled to New York City and Turks and Caicos four months prior, where she reportedly swam in freshwater lakes and walked barefoot on the beach. While she denied consuming raw food or undercooked meats and drinking unfiltered water, she admitted to eating cooked conch and lamb chops. About a month after returning from her travels, the patient developed abdominal pain, diarrhea, and intermittent fevers, initially diagnosed as a UTI and treated with antibiotics. Her symptoms worsened with ongoing abdominal pain, diarrhea, and fevers with rigors, leading to hospitalization. A CT scan in the ED showed indeterminate, geographic areas of low attenuation without discrete lesions or collection, which could reflect hepatitis or small microabscesses as reported in the patient’s history (Figure 1). The patient was started on empirical antibiotics, initially ceftriaxone and metronidazole, and then switched to piperacillin/tazobactam, ertapenem, and micafungin. Despite aggressive therapy, she continued to have daily fevers and leukocytosis.

An extensive infectious workup, including tests for respiratory pathogen panel, gastrointestinal pathogen panel, Clostridioides difficile (C.difficile), human immunodeficiency virus (HIV), Giardia, Entamoeba histolytica, Microsporidia, chronic hepatitis, QuantiFERON-TB Gold, and Aspergillus galactomannan, was negative (Table 1). A Karius test showed exposure to HHV-7 and Parvovirus B19 (IgM-positive). Further tests, including 16S rRNA from a liver biopsy, Echinococcus, Brucella, and Bartonella serologies, were negative. The patient was treated with antibiotics and antifungals and discharged, but her fever persisted.

**Table 1:** Results of an extensive infectious workup all of which returned negative results.

Culture	Result	Reference
HIV	Negative	Negative
Blood	Negative	Negative
Urine	Negative	Negative
RP2	Negative	Negative
Gastrointestinal pathogen panel	Negative	Negative
Amebic antibody	Negative	Negative
Giardia antigen	Negative	Negative
Microsporidia	Negative	Negative
Entamoeba histolytica	Negative	Negative
Chronic hepatitis panel	Negative	Negative
Mononucleosis screen	Negative	Negative
Liver abscess culture	Negative	Negative
Liver abscess AFB	Negative	Negative
Liver abscess fungal	Negative	Negative
Karius HHV-7	Negative	Negative
Parvovirus B19	Positive	Negative
QuantiFERON-TB Gold in-Tube	Negative	Negative
Histoplasma galactomannan urine	Negative	Negative
Aspergillus antigen	Negative	Negative
Fungitell	Negative	Negative
Malaria smear	Negative	Negative
Leptospira antibody	Negative	Negative
Echinococcus antibody	Negative	Negative
Brucella antibody IgM	Negative	< 1:80
Bartonella henselae Antibody, IgG	<1:64	< 1:128
Bartonella henselae Antibody, IgM	<1:16	< 1:20
Bartonella quintana Antibody, IgG	<1:64	< 1:128
Bartonella quintana antibody, IgM	<1:16	< 1:20
Coxiella burnetti IgG, Phase 1	Negative	< 1:16
Coxiella burnetti IgG, Phase 2	Negative	< 1:16
Coxsackie B Type 1	<1:10	< 1:10
Coxsackie B Type 2	<1:10	< 1:10
Coxsackie B Type 3	1:320	< 1:10
Coxsackie B Type 4	1:160	< 1:10
Coxsackie B Type 5	1:10	< 1:10
Coxsackie B Type 6	<1:10	< 1:10
p-ANCA fluorescence	Negative	< 1:20
Rheumatoid factor	<10 U/mL	< 20 U/mL
C3	176 mg/dL	88-210 mg/dL
C4	29 mg/dL	15-45 mg/dL

A month later, the patient returned with persistent low-grade fevers (100-102°F) and vomiting. An extensive infectious workup was negative. The differential diagnosis included aseptic abscess syndrome related to inflammatory bowel disease and extrapulmonary sarcoidosis. Given the suspicion of autoimmune involvement, she was started on prednisone 40 mg daily and advised to follow up with a rheumatologist. At her last outpatient infectious disease visit, her fever had resolved, and no formal diagnosis was made. She continues outpatient evaluation for potential sarcoidosis or SLE.

## Discussion

This case presents a complex diagnostic challenge involving a young female patient with multiple comorbidities and a prolonged FUO despite an extensive workup. The patient’s history of thrombophilia, obesity, and pituitary adenoma, combined with her recent travel to an endemic area, raised concerns for a variety of infectious, autoimmune, and inflammatory causes. The differential diagnosis for FUO is broad, encompassing infections, malignancies, and autoimmune conditions. In this case, an extensive infectious workup, including tests for common and rare pathogens (C. difficile, Giardia, Entamoeba histolytica, HIV, and others), returned negative results, with the exception of exposure to HHV-7 and Parvovirus B19. This suggested that infectious causes might be less likely, although the role of these viral exposures in the patient's clinical presentation remains uncertain. Infections such as brucellosis, Bartonella, and Echinococcus were considered, with pending serologies at the time of discharge, but no definitive diagnosis was made. Hepatic abscesses, which were identified on a CT scan, are most commonly caused by bacterial infections, but can also result from parasitic infections or noninfectious causes, including malignancy and autoimmune disease. In this case, despite appropriate antimicrobial therapy, the patient’s persistent fever and leukocytosis pointed to the possibility of an underlying inflammatory or autoimmune process. Extrapulmonary sarcoidosis and aseptic abscess syndrome, particularly in the context of inflammatory bowel disease, were considered as part of the differential diagnosis. Sarcoidosis, a systemic granulomatous disease, can present with hepatic involvement and abscess formation, although it is a rare cause of such findings [8]. The use of corticosteroids (prednisone) was initiated due to concerns about inflammation, particularly given the patient's unresolved symptoms and the possibility of an autoimmune process. Steroids are often used in the treatment of both sarcoidosis and autoimmune diseases like SLE, which can also manifest with a similar array of nonspecific symptoms, including fever, diarrhea, and weight loss [9,10]. The role of autoimmune diseases, including SLE, was further emphasized by the patient's ongoing clinical course and lack of response to antibiotics. SLE is known to present with systemic symptoms such as fever, weight loss, and organ involvement, including the liver, and can lead to vasculitis and abscess formation [10]. Given the patient's complex presentation and the potential for autoimmune involvement, referral to a rheumatologist was recommended to further evaluate the possibility of SLE or another autoimmune condition. This case highlights the importance of a comprehensive approach to FUO, considering both infectious and noninfectious etiologies. While infectious causes were ruled out, autoimmune diseases like sarcoidosis and SLE remain high on the differential and warrant ongoing evaluation. It also underscores the utility of multidisciplinary collaboration in managing complex cases, involving both infectious disease specialists and rheumatologists to reach a diagnosis and guide treatment.

## Conclusions

Our case presentation demonstrates the critical need to consider autoimmune diseases, such as SLE, in the differential diagnosis of FUO, particularly after infectious etiologies are excluded. Lupus-associated granulomatous hepatitis is a rare and challenging manifestation of SLE that can mimic infectious processes, making prompt recognition critical for appropriate management. Liver biopsy and serological testing played a crucial role in our case in identifying the underlying autoimmune etiology. Early initiation of corticosteroid therapy also led to significant clinical improvement here which demonstrates the importance of a systematic, multidisciplinary approach to diagnosing and treating FUO.
